# Liraglutide ameliorates oxidized LDL-induced endothelial dysfunction by GLP-1R-dependent downregulation of LOX-1-mediated oxidative stress and inflammation

**DOI:** 10.1080/13510002.2023.2218684

**Published:** 2023-06-06

**Authors:** Wu Ying, Song Meiyan, Chen Wen, Xu Kaizu, Wu Meifang, Lin Liming

**Affiliations:** Department of Cardiology, Affiliated Hospital of Putian University, Department of Clinical Medicine, Fujian Medical University, Putian, People’s Republic of China

**Keywords:** Liraglutide, oxidized low-density lipoprotein, lectin-like ox-LDL receptor-1, endothelial dysfunction, oxidative stress, inflammation

## Abstract

**Objective:**

To investigate the effects of glucagon-like peptide 1 receptor (GLP-1R) agonist liraglutide on endothelial dysfunction in LDL receptor-deficient (LDLR-KO) mice and ox-LDL-challenged human umbilical vein endothelial cells (HUVECs) and its possible mechanism.

**Methods:**

LDLR-KO mice were randomly treated with normal saline, liraglutide, or liraglutide plus a GLP-1R antagonist exendin-9 for four weeks. In parallel, HUVECs were cultured with ox-LDL alone or combined with liraglutide, in the presence or absence of lectin-like ox-LDL receptor-1(LOX-1) overexpression or GLP-1R knockdown. Endothelial-dependent relaxation and LOX-1 protein expression of thoracic aorta, circulating levels of oxidative and inflammatory markers in mice, and cell survival, reactive oxygen species production, and expression of adhesion molecules and signal regulators in ox-LDL cultured endothelial cells were measured.

**Results:**

liraglutide effectively enhanced acetylcholine-induced vasodilation, reduced LOX-1 expression in aortas, and decreased circulatory oxidative and inflammatory levels in LDLR-KO mice, which were abolished by cotreatment with exendin-9. HUVECs exposed to ox-LDL exhibited reduced cell viability, increased reactive oxygen species production and apoptosis, and elevated protein expression of ICAM-1, VCAM-1, LOX-1, NOX4, and NF-κB, which were markedly ameliorated by liraglutide treatment. The protective effects of liraglutide against ox-LDL-induced cell injury were abrogated in HUVECs overexpressing LOX-1 or silencing GLP-1R.

**Conclusions:**

Liraglutide improved oxidized LDL-induced endothelial dysfunction via GLP-1R-dependent downregulation of LOX-1-mediated oxidative stress and inflammation.

## Introduction

Atherosclerotic cardiovascular disease (ASCVD) is the leading cause of death and disability worldwide [1]. Endothelial dysfunction, characterized by excessive oxidative stress levels, upregulation of proinflammatory cytokines and chemokines, increased endothelial apoptosis, and reduced bioavailability of nitric oxide (NO), is considered the initial step in atherosclerosis development [2,3], and is associated with accelerated atherosclerosis in chronic immune-mediated diseases [4,5]. Liraglutide, a glucagon-like peptide 1 receptor (GLP-1R) agonist, is a novel hypoglycemic agent recommended by the latest guidelines for treating type 2 diabetes and comorbid ASCVD due to its proven cardiovascular benefits [6–8]. The mechanisms responsible for liraglutide reducing ASCVD events are multifactorial, among which its direct protective effect on vascular endothelium has gained intense interest recently and has been suggested to be independent of its hypoglycemic effect [9]. Moreover, the widespread distribution of GLP-1R in the vascular system, including endothelial cells, smooth muscle cells, and macrophages [10,11], provides further evidence for the potential direct protective effect of liraglutide against atherosclerosis. Helmstadter et al. found that liraglutide can improve cardiovascular remodeling and function, as well as reduce vascular inflammation and oxidative stress in hypertensive mice through the activation of GLP-1R in endothelial cells [12]. Gaspari et al. discovered that liraglutide enhanced vascular reactivity in ApoE-/- mice, which was accompanied by upregulated expression of eNOS and downregulated expression of vascular adhesion molecules [13]. However, the precise signaling pathway underlying the beneficial effects of liraglutide on vascular endothelial dysfunction, inflammation, and oxidative stress remains elusive.

Oxidized low-density lipoprotein (ox-LDL) is the oxidative modification of LDL triggered by various cardiovascular risk factors and plays a crucial role in the initiation and progression of atherosclerosis through binding to its central mediator, lectin-like ox-LDL receptor-1 (LOX-1) [14,15]. Generally, ox-LDL induces up-regulation of LOX-1 expression in endothelial cells, and binds to it to increase NADPH oxidase (NOX, mainly NOX-4)-derived reactive oxygen species (ROS) generation. The increased ROS then disrupts endothelial nitric oxide synthase decoupling and NO synthesis, activates the downstream nuclear factor κB (NF-κB), and increases the expression of pro-inflammatory cytokines such as monocyte chemoattractant protein-1, intercellular adhesion molecule-1 (ICAM-1), and vascular intercellular adhesion molecule-1 (VCAM-1) in endothelial cells, which results in enhanced leukocyte adhesion and endothelial apoptosis [16–18]. Therefore, blocking ox-LDL-induced endothelial dysfunction mediated by the LOX-1/NOX-4/ NF-κB pathway may serve as a therapeutic strategy for the treatment of ASCVD.

In recent years, mounting evidence has reported the protective effects of GLP-1R agonists against endothelial dysfunction [19–21]. However, few articles have investigated the effects of GLP-1R on ox-LDL-induced LOX-1-mediated endothelial dysfunction. In vascular smooth muscle cells treated with ox-LDL, the upregulation of LOX-1 expression serves as a crucial mediator in ROS production and is also involved in the downregulation of GLP-1R expression. Liraglutide effectively reduces ROS production induced by ox-LDL. Conversely, anti-LOX-1 antibodies can reverse GLP-1R inhibition caused by ox-LDL, while overexpression of LOX-1 can alleviate liraglutide’s inhibitory effect on ROS production [22]. Therefore, in this study, we aimed to investigate the effects of liraglutide on endothelial dysfunction in LDL receptor-deficient mice and ox-LDL-challenged human umbilical vein endothelial cells (HUVECs) and to explore the role of GLP-1R and LOX-1 in this process.

## Methods

### Animal grouping and treatment

Eight-week-old male wild-type (C57BL/6 mice) and homozygous LDL receptor-deficient (LDLR-KO) mice were purchased from the Shanghai SLAC Laboratory Animal Co. Ltd. The LDLR-KO mice were adapted to a high cholesterol (4% cholesterol/10% cocoa butter) diet for six weeks and then randomly assigned to receive the following treatment for four weeks: normal saline (LDL-KO group, *n* = 10), liraglutide (LDL-KO + LIR group, liraglutide 300 mg/kg, twice daily, s.c, *n* = 10), or liraglutide plus a GLP-1R antagonist exendin-9 (Bachem Bioscience, King of Prussia, PA) (LDL-KO + LIR + EXE group, liraglutide 300 mg/kg, twice daily, s.c, and exendin-9 150 pmol/kg per min via mini osmotic pump). C57BL/6 mice received a standard chow diet and normal saline treatment. All animals were housed in temperature-controlled cages (20–22°C), fed ad libitum, and maintained on a 12:12 h light/dark cycle. All the experiments were approved by the Animal Ethics Committee of the Affiliated Hospital of Putian University and performed following institutional guidelines.

### Organ chamber experiments

Endothelium-dependent vasodilation was assessed by organ chamber experiments as described previously [23]. In brief, the aorta was cleaned from adhering connective tissue under a dissection microscope and cut into 2 mm rings for organ chamber experiments. Each ring was then connected to an isometric force transducer (PowerLab ML870 8/30, AD Instruments, Bella Vista, Australia), suspended in an organ chamber filled with 5 mL Krebs–Ringer bicarbonate solution (37°C, pH 7.4), and bubbled with 95% O_2_ and 5% CO_2_. Concentration-effect curves were produced by adding graded concentrations of acetylcholine (10^−9^–10^−5^ mol/L) during submaximal contractions to norepinephrine (10^−5^ mol/L). Relaxations were expressed as percentages of precontracted tension and pD2 values (−1og10 EC50) for acetylcholine and interpolated from each curve.

### Circulatory levels of ox-LDL, SOD, MDA, IL-1β, IL-6, and NO

The plasma samples were separated from the blood. The plasma levels of ox-LDL, interleukin-1β (IL-1β), and interleukin-6 (IL-6) were detected using an enzyme-linked immunosorbent assay (ELISA) kit (Jiancheng Biotech Co. Ltd., Nanjing, China). The plasma levels of malondialdehyde (MDA) were determined by the thiobarbituric acid (TBA) method according to the MDA assay kit (Jian Cheng Biotechnology Co., Ltd., Nanjing, China) specifications. The absorbance values used for the MDA measurements were obtained at 532 nm by a microplate reader and converted to nmol/L [24]. The superoxide dismutase (SOD) activity was detected by WST-1 assay using the SOD assay kit (Jian Cheng Biotechnology Co., Ltd., Nanjing, China), as described previously [25]. The absorbance values used for the measurements of SOD activity in serum were obtained at 450 nm by a microplate reader and converted to U/ml. NO levels were measured by the Griess method using a nitric oxide detection kit (Enzo Life Sciences).

### Cell culture and treatment

HUVECs were isolated as described previously [23]. Cells were seeded in DMEM supplemented with 10% fetal bovine serum, 100 U/mL penicillin, and 100 μg/mL streptomycin sulfate, and maintained in a humidified incubator with 5% CO_2_ at 37°C. HUVECs between passages 3 and 5 were used for the experiment. For the treatment group (ox-LDL + Liraglutide group), the cells were pretreated with 1000 nM liraglutide (Novo Nordisk Pharmaceutical Co., Ltd.) for 1 h, followed by exposure to ox-LDL (LM-G5689, Shanghai Lianmai Bio) at a concentration of 20 µg/mL (µg protein/mL of solution) for 24 h. The degree of ox-LDL oxidation was assessed using the thiobarbituric acid reactivity assay, as described by Ohkawa et al. [26]. Briefly, MDA served as a standard, and thiobarbituric acid-reactive substances (TBAR) values were expressed as equivalents of MDA per milligram of LDL protein. The oxidized LDL utilized in this experiment exhibited a TBAR value of 3.84. All the experimental operations were approved by the Ethics Committee of the Affiliated Hospital of Putian University and were performed following the Declaration of Helsinki.

### Cell transfection

HUVECs were transfected with pcDNA3.1 null control or pcDNA3.1-LOX-1 (5 μg) (DP103-02, Tiangen Biotech), respectively, by using lipofectamine 2000 (L3000015, Invitrogen™). Immunoblotting was performed to determine the transfection efficiency.

### Cell viability assay

The CCK-8 assay (KGA317, Kekgen Biotech) was used to investigate the cell viability of HUVECs cultured in 96-well plates. In brief, after different treatments, 100 μl CCK-8 solution at a 1:10 dilution was added to each well of the plate followed by a further 2–4 h incubation in the incubator. Absorbance was measured at 450 nm with a microplate reader (Molecular Devices, Sunnyvale, CA, USA). The mean optical density of five wells in the indicated groups was used to calculate the cell viability percentage. Experiments were performed in triplicate.

### Apoptosis detection by flow cytometry

For apoptosis detection, 1 × 10^6^ to 3 × 10^6^ cells were collected, centrifuged with 1 mL of PBS at 1500 rpm for 3 min, and washed twice. Next, 5x Binding Buffer was diluted with double distilled water to 1x Binding Buffer. Subsequently, the cells were resuspended in 300 µl of precooled 1x Binding Buffer. Annexin V-FITC (5 µl) and PI (10 µl) were then added to each tube. The labeling agents were mixed gently, and the cells were incubated in the dark for 10 min at room temperature. Subsequently, 200 µl of precooled 1x Binding Buffer was added to each tube and mixed well for detection by flow cytometry.

### Detection of cellular ROS activity by flow cytometry

DCFH-DA was diluted with a serum-free medium at 1:1000 to a final concentration of 10 µmol/l. The cell culture medium was discarded, and the diluted DCFH-DA solution was then added to the cells and incubated for 20 min at 37°C. The cells were then washed three times to remove excess DCFH-DA. Subsequently, the cells were washed with 1 mL of PBS and centrifuged at 1500 rpm for 5 min, and the supernatant was discarded. The cells were resuspended in 300 µl PBS and detected by flow cytometry.

### Determination of ICAM-1 and VCAM-1 levels in the medium

The conditioned medium was collected and the ICAM-1 and VCAM-1 levels were determined using ELISA kits (R&D Systems, Minneapolis, MN, USA) according to the manufacturer’s instructions.

### Immunoblotting

For immunoblotting, protein extracts (15 µg) from the thoracic aorta or total cellular proteins (50 µg) were electrophoresed on 10% SDS-PAGE and transferred onto nitrocellulose membranes. The membranes were blocked with 5% dry milk in PBS-Tween buffer (0.1% Tween 20; pH 7.5) for 60 min and incubated with mouse monoclonal anti-GAPDH (TA-08, Zhongqian Jinqiao, 1/2000), rabbit anti-LOX-1 (alias: OLR1, 11837-1-ap, Proteintech, 1/1000), rabbit anti-NOX4 (Millipore, Billerica, MA, USA, 1/2000), rabbit polyclonal anti-NFĸB p65 (Santa Cruz Biotechnology, CA, USA, 1/1000); horseradish peroxidase-labeled goat anti-mouse IgG (H + L) (ZB-2305, Zhongshan Jinqiao, 1/2000), horseradish peroxidase-labeled goat anti-rabbit IgG (H + L) (ZB-2301, Zhongqian Jinqiao, 1/2000). The immunoreactive bands were detected by an enhanced chemiluminescence system (Millipore, Billerica, USA). Related signals were quantified using Scion Image Software (Scion Corp., Frederick, USA).

### RNA silencing by small interfering RNA (siRNA)

The sense and antisense human GLP-1R siRNAs used in this experiment (5′-UCAUCAAGCUGUUUACAGAtt-3′ and 5′-UCUGUAAACAGC UUGAUGAag-3′, respectively) were synthesized by Applied Biosystems (Foster, CA, USA). Scrambled non-silencing siRNAs were also obtained from Applied Biosystems (Silencer Negative Control #1 siRNA). Then the siRNA duplexes were transfected to HUVEC using Lipofectamine™ 2000 (Invitrogen, Carlsbad, CA, USA) according to the manufacturer’s recommendations. After two days of transfection, GLP-1R protein was analyzed.

### Statistical analyses

SPSS 20.0 software was used for statistical analyses. The quantitative results were expressed as mean ± *SE*. One-way ANOVA was used to compare quantitative values among the multiple groups, and the S-N-K method was used for two-way comparisons. The differences between the groups were considered to be significant at *α* = 0.05 and *P *< 0.05.

## Results

### Liraglutide enhanced acetylcholine-induced vasorelaxation in LDLR-KO mice in a GLP-1R-dependent manner

**Figure 1.**
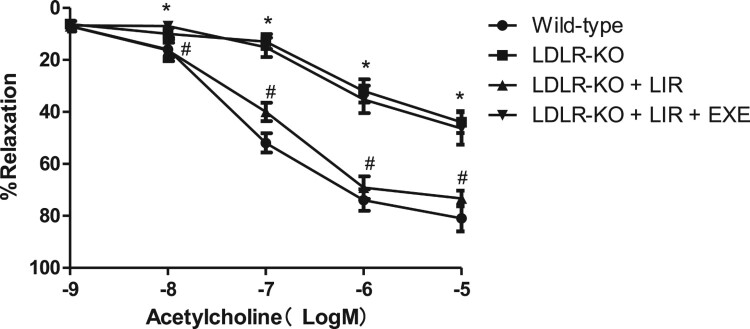
Effects of liraglutide on the acetylcholine-induced relaxation of the aorta from LDLR-KO mice and the role of exendin-9. After 1 h of incubation, the aortic rings were precontracted with norepinephrine (10^−5^ M), and then the rings were exposed to a cumulative concentration of acetylcholine (10^−9^–10^−5^ M) to test the endothelial-dependent vasodilation. LIR indicates liraglutide. EXE indicates exendin-9. Results are mean ± *SE*, *n* = 4 in each group, *P < 0.05 compared to wild-type at the same concentration, ^#^*P *< 0.05 compared to LDLR-KO at the same concentration.

As shown in Figure 1, the endothelium-dependent vasorelaxation to acetylcholine (10^−9^–10^−5^ M) of the aortas from vehicle-treated LDLR-KO mice was markedly impaired as compared to those from wild-type mice; when acetylcholine was at concentrations greater than or equal to 10^−8^ M, the diastolic response was significantly different between the two groups (*P* < 0.05). Liraglutide treatment significantly improved endothelium-dependent relaxation compared with vehicle-treated LDLR-KO mice (*P* < 0.05). The pD2 values in the liraglutide-treated LDLR-KO mice were significantly higher than those in the vehicle-treated LDLR-KO mice (7.02 ± 0.18 vs. 6.44 ± 0.35, *P *< 0.05). However, the coadministration of liraglutide and GLP-1R antagonist exendin-9 abolished the protection by liraglutide.

### Liraglutide alleviated circulatory oxidative and inflammatory markers in LDLR-KO mice in a GLP-1R-dependent manner

**Figure 2.**
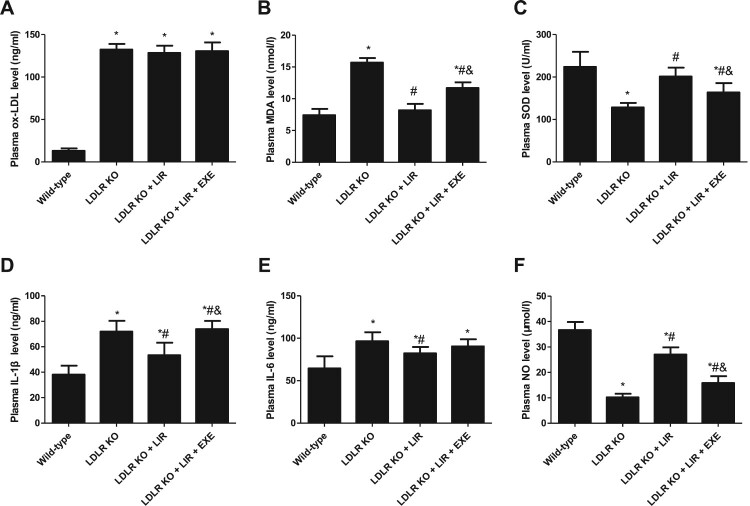
Effects of liraglutide on the circulatory levels of ox-LDL, oxidative and inflammatory markers, and NO bioavailability in LDLR-KO mice and the role of exendin-9. LIR indicates liraglutide. EXE indicates exendin-9. Results are mean ± *SE*, *n* = 10 in each group, *P < 0.05 compared to wild-type mice, ^#^*P *< 0.05 compared to LDLR-KO mice, ^&^*P *< 0.05 compared to liraglutide-treated LDLR-KO mice.

As shown in Figure 2(A), the plasma levels of ox-LDL in LDLR-KO mice were remarkably higher than those in wild-type mice. Both liraglutide and liraglutide plus exendin-9 failed to reduce ox-LDL levels in LDLR-KO mice. The plasma levels of MDA, a broadly used marker of lipid peroxidation, were significantly elevated in LDLR-KO mice compared to those in wild-type mice, which were significantly attenuated by liraglutide treatment. Co-administration of liraglutide with exendin-9 partially counteracts the antioxidant effects of liraglutide (Figure 2(B)). SOD, a crucial endogenous antioxidant enzyme, functions as a component of the primary line of defense against ROS. The plasma levels of SOD were significantly decreased in LDLR-KO mice compared to those in wild-type mice, which were partially restored by liraglutide treatment. Similarly, when used in combination with exendin-9, the liraglutide’s antioxidant-elevating effect was diminished (Figure 2(C)). Moreover, the elevated circulatory levels of pro-inflammatory cytokines IL-1β and IL-6, as well as reduced NO levels, in LDLR-KO mice were also ameliorated by liraglutide, and these effects were also attenuated after treatment with exendin-9 (Figure 2(D–F)).

### Liraglutide decreased LOX-1 expression in the thoracic aorta from LDLR-KO mice in a GLP-1R-dependent manner

**Figure 3.**
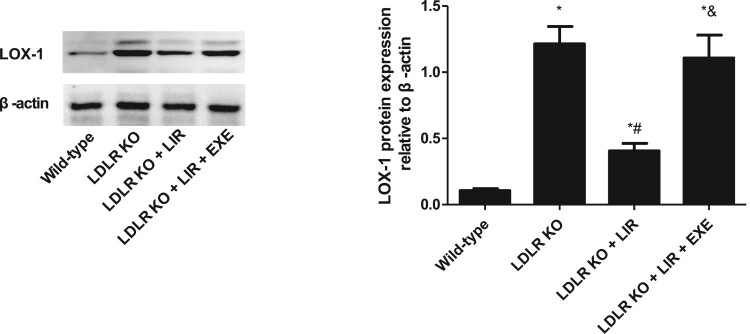
Effect of liraglutide on the LOX-1 expression in the thoracic aorta from LDLR-KO mice and the role of exendin-9. LIR indicates liraglutide. EXE indicates exendin-9. The results of three independent experiments were expressed as mean ± *SE*. *P < 0.05 compared to wild-type mice, ^#^*P *< 0.05 compared to LDLR-KO mice, ^&^*P *< 0.05 compared to liraglutide-treated LDLR-KO mice.

Immunoblotting of LOX-1 in the aorta was used to compare the LOX-1 expression among different groups. As shown in Figure 3, the LOX-1 protein expression in the thoracic aorta from vehicle-treated LDLR-KO mice was remarkably higher than those from wild-type mice, which was significantly attenuated by liraglutide treatment. However, the inhibitory effect of liraglutide on LOX-1 expression was abolished when LDLR-KO mice were given liraglutide plus exendin-9 treatment.

### Liraglutide ameliorated ox-LDL-induced LOX-1-mediated cytotoxicity

Firstly, cells were treated with ox-LDL 0 µg/mL, 10 µg/mL, 20 µg/mL, 40 µg/mL, or 80 µg/mL, respectively, for 24 h, and then intracellular ROS levels were determined by flow cytometry. The results showed that the fluorescence intensity reached its peak when the ox-LDL concentration was 20 µg/mL. Afterward, with the increase of ox-LDL concentration, intracellular ROS levels decreased (Figure 4(A)). Therefore, 20 µg/mL ox-LDL was used for the subsequent experiments. Similarly, cells were treated with liraglutide 10–1000 nM for 24 h, and then cell viability was measured by CCK-8 assay to determine the optimal work concentration of liraglutide. As shown in Figure 4(B), with the increase in liraglutide concentration, the cell survival rate tended to increase, and liraglutide at 1000 nM showed significantly higher cell survival than the control group. Therefore, 1000 nM liraglutide was selected for the subsequent experiments. Figure 4.Effect of liraglutide on ox-LDL-induced LOX-1-mediated cytotoxicity. (A) cells were treated with ox-LDL 0, 10, 20, 40, or 80 µg/ml, respectively, for 24 h and then intracellular ROS levels were determined by flow cytometry. The results of three independent experiments were expressed as mean ± *SE*. *P < 0.05 compared to control, ^#^*P *< 0.05 compared to ox-LDL 10 µg/ml. (B) Cells were treated with liraglutide 10–1000 nM for 24 h and then cell viability was measured by CCK-8 assay. The results of three independent experiments were expressed as a percentage relative to the control. *P < 0.05 compared to control. (C) Cells were transfected with pcDNA3.1 null control or pcDNA3.1-LOX-1, respectively, and LOX-1 protein expression was determined by immunoblotting. The results of three independent experiments were expressed as a fold of control. *P < 0.05 compared to control. (D) Cells transfected with pcDNA3.1 null control or pcDNA3.1-LOX-1 were treated with 20 µg/mL ox-LDL alone or combined with 1000 nM liraglutide and cell viability was determined by CCK-8 assay. The results of three independent experiments were expressed as a percentage relative to the control. *P < 0.05 compared to control, ^#^*P *< 0.05 compared to ox-LDL group, ^&^*P *< 0.05 compared to ox-LDL + liraglutide group. (E) Cells transfected with pcDNA3.1 null control or pcDNA3.1-LOX-1 were treated with ox-LDL alone or combined with liraglutide 1000 nM and cell apoptosis was determined by flow cytometry. The results of three independent experiments were expressed as mean ± SE. *P < 0.05 compared to control, ^#^*P *< 0.05 compared to ox-LDL group, ^&^*P *< 0.05 compared to ox-LDL + liraglutide group.

**Figure:**
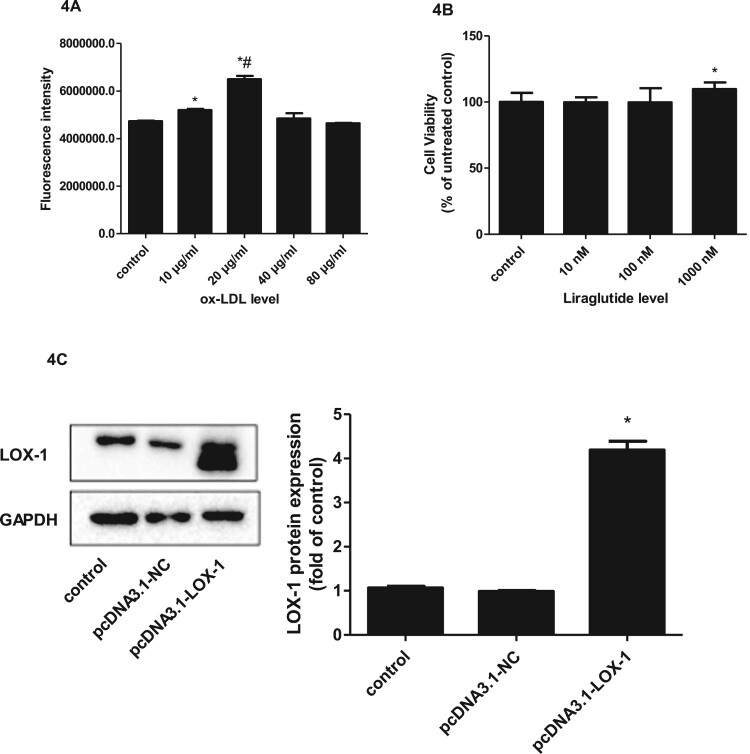

**Figure:**
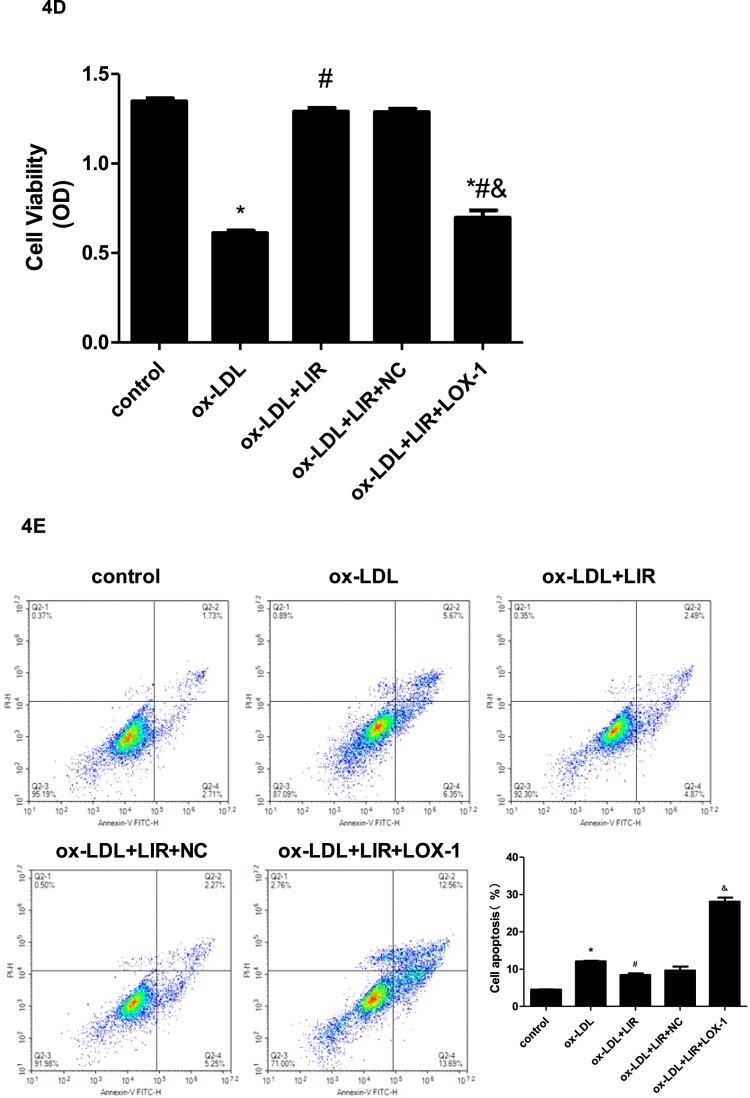

Secondly, cells were transfected with pcDNA3.1 null control (NC) or pcDNA3.1-LOX-1, respectively, and an immunoblotting assay was performed to determine the transfection efficiency. As shown in Figure 4(C), the relative protein expression of LOX-1 in cells transfected with pcDNA3.1-LOX-1 was significantly higher than those transfected with pcDNA3.1 null control.

After cell transfection, we evaluated the effects of liraglutide on ox-LDL-induced cytotoxicity and the role of LOX-1 in this process. As shown in Figure 4(D), cells treated with ox-LDL showed reduced cell viability compared with those treated with a control medium, whereas cells pretreated with liraglutide and then exposed to ox-LDL showed remarkably restored cell viability as compared to those treated with ox-LDL alone. However, the protective effect of liraglutide on ox-LDL-induced cytotoxicity was abrogated in cells overexpressing LOX-1. Then, we further detected the apoptosis rate by flow cytometry. Similarly, as shown in Figure 4(E), the ox-LDL challenge significantly increased cell apoptosis compared to the control medium, and liraglutide pretreatment effectively attenuated the increased apoptosis induced by ox-LDL. When cells were pre-transfected with pcDNA3.1-LOX-1, the inhibitory effect of liraglutide on ox-LDL-induced apoptosis was considerably reversed.

### Liraglutide reduced ox-LDL-induced LOX-1-mediated ROS generation

**Figure 5.**
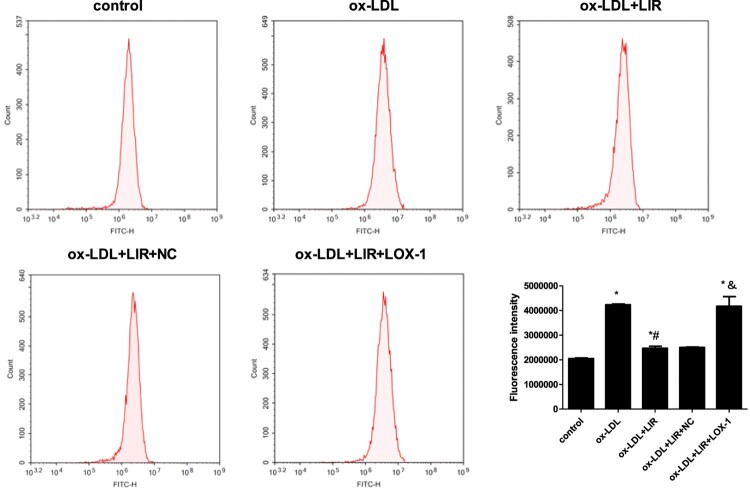
Effect of liraglutide on ox-LDL-induced LOX-1-mediated ROS generation. Cells transfected with pcDNA3.1 null control or pcDNA3.1-LOX-1 were treated with 20 µg/mL ox-LDL alone or combined with 1000 nM liraglutide and intracellular ROS levels were determined by flow cytometry. The results of three independent experiments were expressed as mean ± *SE*. *P < 0.05 compared to control, ^#^*P *< 0.05 compared to ox-LDL group, ^&^*P *< 0.05 compared to ox-LDL + liraglutide group.

Excess oxidative stress is considered to be a major driver of ox-LDL-induced cytotoxicity, and then the intracellular ROS generation was measured by flow cytometry. As shown in Figure 5, cells exposed to ox-LDL treatment alone presented significantly higher ROS levels than those treated with a control medium, whereas cells pretreated with liraglutide and then exposed to ox-LDL treatment showed a dramatic reduction in ROS generation as compared to those in the ox-LDL group. Similarly, when cells were transfected with LOX-1 in advance, the inhibitory effect of liraglutide on ox-LDL-induced ROS generation was strikingly impaired.

### Liraglutide attenuated ox-LDL-induced LOX-1-mediated upregulation of ICAM-1 and VCAM-1

**Figure 6.**
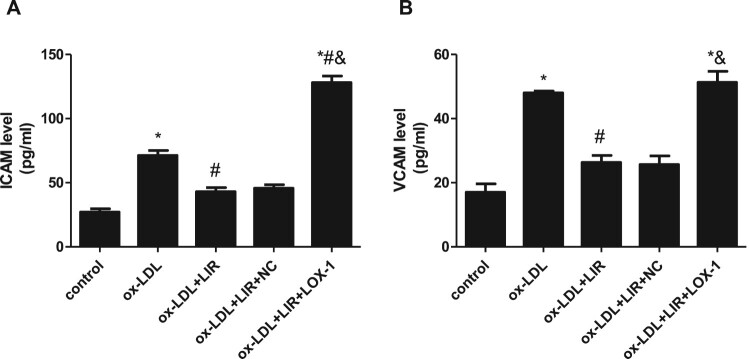
Effect of liraglutide on ox-LDL-induced LOX-1-mediated upregulation of ICAM-1 and VCAM-1. Cells transfected with pcDNA3.1 null control or pcDNA3.1-LOX-1 were treated with 20 µg/mL ox-LDL alone or combined with 1000 nM liraglutide and ICAM-1 and VCAM-1 level was determined by ELISA assay. The results of three independent experiments were expressed as mean ± *SE*. *P < 0.05 compared to control, ^#^*P *< 0.05 compared to ox-LDL group, ^&^*P *< 0.05 compared to ox-LDL + liraglutide group.

Aberrant adhesion molecules secretion is another phenotype of endothelial dysfunction, which drives increased monocyte chemotaxis and migration to subintimal space and phagocytosis of ox-LDL and finally aggregates atherosclerosis development. Given that, the ICAM-1 and VCAM-1 levels in the supernatant of the cultures were detected by ELISA assay. As shown in Figures 6(A,B), both the levels of ICAM-1 and VCAM-1 were significantly increased in the ox-LDL group as compared to those in the control group. Liraglutide pretreatment almost completely normalized the upregulation of ICAM-1 and VCAM-1 induced by ox-LDL. However, the ability of liraglutide to inhibit ICAM-1 and VCAM-1 secretion was dramatically compromised when cells were transfected with pcDNA3.1-LOX-1 in advance.

### Liraglutide blocked ox-LDL-induced activation of the LOX-1/NOX4/NF-κB pathway in a GLP-1R-dependent manner

NADPH oxidase 4 (NOX4) is the most highly expressed enzyme in vascular endothelial cells than other NADPH oxidase family members and is responsible for ROS generation and reduced NO bioavailability [27]. The increased ROS production then activates NF-κB signaling in ox-LDL-treated endothelial cells to promote further inflammation [17]. For these reasons, the protein expression levels of LOX-1, NOX4, and NF-κB p65 were determined by immunoblotting. As shown in Figure 7(A), the protein expression levels of LOX-1, NOX4, and NF-κB p65 in the ox-LDL group were strikingly increased as compared to those in the control group (*P *< 0.05). Liraglutide significantly inhibited the upregulated expression of LOX-1, NOX4, and NF-κB p65 induced by ox-LDL (*P *< 0.05). However, the ability of liraglutide to inhibit LOX-1, NOX4, and NF-κB protein expression was reduced in cells transfected with pcDNA3.1-LOX-1 (Figure 7(A)). Figure 7.Effect of liraglutide on ox-LDL-induced activation of LOX-1/NOX4/NF-κB pathway and the role of GLP-R. (A) Cells transfected with pcDNA3.1 null control or pcDNA3.1-LOX-1 were treated with ox-LDL alone or combined with liraglutide 1000 nM and protein expression levels of LOX-1, NOX4, and NF-κB p65 were determined by immunoblotting. The results of three independent experiments were expressed as mean ± *SE*. *P < 0.05 compared to control, ^#^*P *< 0.05 compared to ox-LDL group, ^&^*P *< 0.05 compared to ox-LDL + liraglutide group. (B) Cells were pretreated with the NOX-4 inhibitor (GKT137831, 10 μmol/l) for 1 h and then exposed to 20 µg/ml ox-LDL for 24 h. The protein expression of NF-κB p65 was determined by immunoblotting. The results of three independent experiments were expressed as mean ± *SE*. *P < 0.05 compared to control, ^#^*P *< 0.05 compared to ox-LDL group. (C) Cells were transfected with scrambled siRNA or GLP-1R siRNA, respectively, and GLP-1R protein expression was determined by immunoblotting. The results of three independent experiments were expressed as a fold of control. *P < 0.05 compared to control. (D) Cells transfected with scrambled siRNA or GLP-1R siRNA were treated with ox-LDL alone or combined with liraglutide, and then the protein expression of LOX-1 was determined by immunoblotting. The results of three independent experiments were expressed as mean ± *SE*. *P < 0.05 compared to control, ^#^*P *< 0.05 compared to ox-LDL group, ^&^*P *< 0.05 compared to ox-LDL + liraglutide group.

**Figure:**
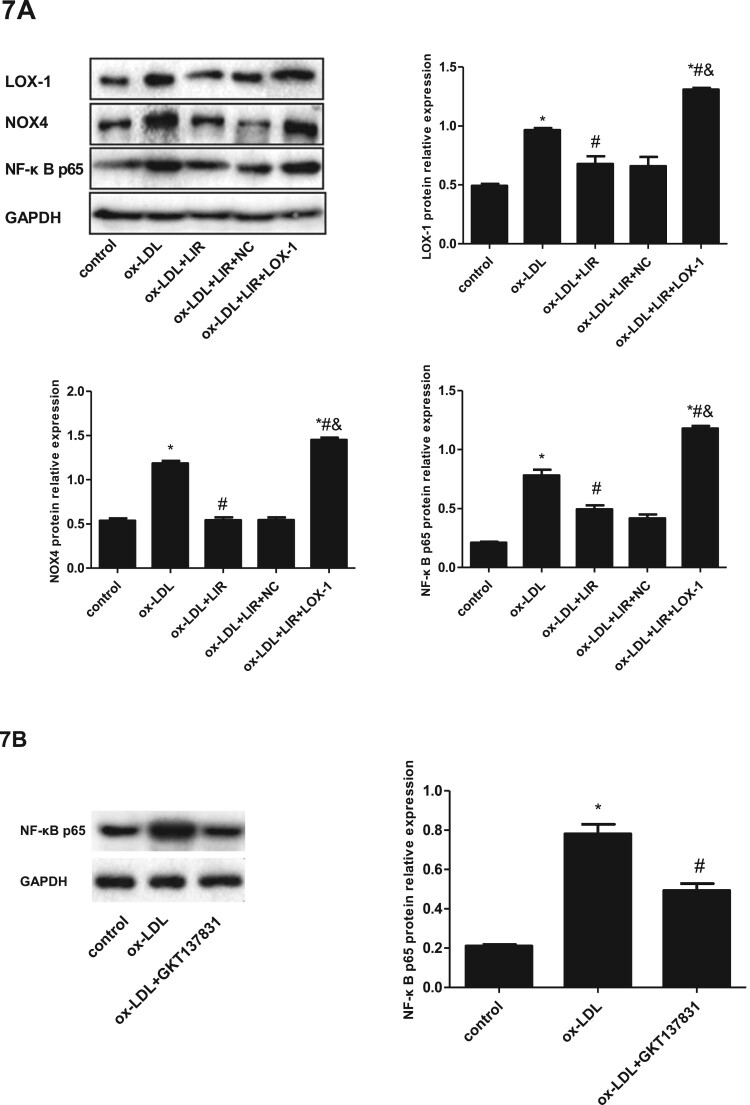

**Figure:**
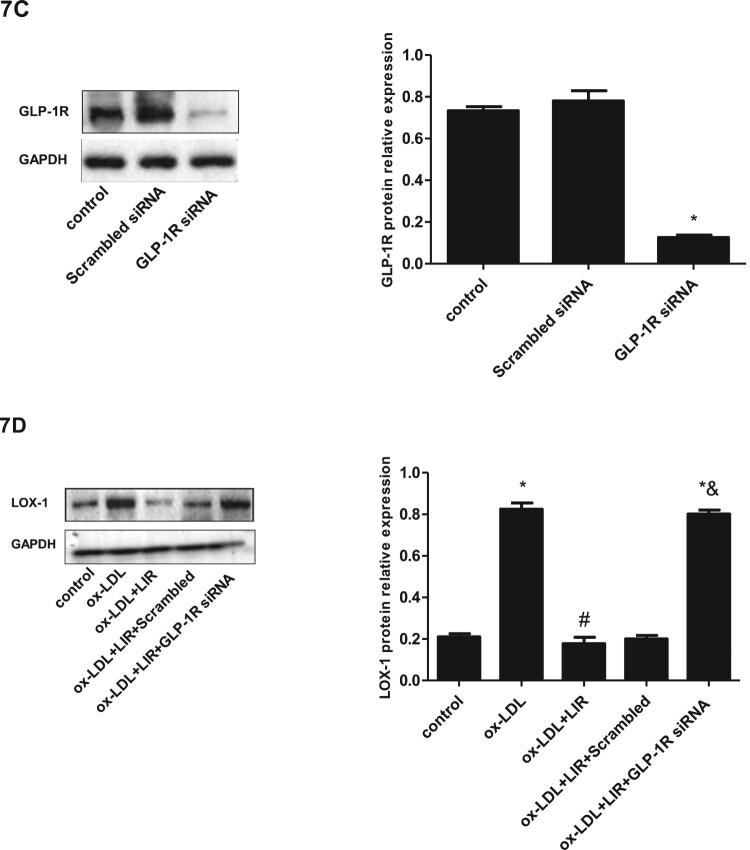

To further explore whether NOX4 is the upstream regulator of NF-κB p65 in ox-LDL-cultured HUVECs, cells were treated with ox-LDL alone or combined with NOX4 inhibitor GKT137831. We found that the upregulated expression of NF-κB p65 in ox-LDL-cultured HUVECs was significantly mitigated by NOX4 inhibitor GKT137831, suggesting that NOX4 contributes to NF-κB p65 upregulation in ox-LDL-culture HUVECs (Figure 7(B)).

At last, we determined the role of GLP-1R in the downregulation of LOX-1 protein expression by liraglutide in ox-LDL-cultured HUVECs. Cells transfected with GLP-1R siRNA showed significantly reduced expression of GLP-1R protein compared to those transfected with scrambled siRNA (Figure 7(C)). Moreover, the LOX-1 protein expression in cells transfected with siRNA against GLP-1R presented a nonsignificant change in response to liraglutide treatment, suggesting that GLP-1R is essential for the downregulation of LOX-1 in endothelial by liraglutide (Figure 7(D)).

## Discussion

A growing body of evidence has demonstrated the extra cardiovascular benefits of GLP-1R agonists beyond the glucose-lowering effect, however, the underlying mechanisms are still not fully understood. In addition, the extensive expression of GLP-1R in the vascular system has also prompted researchers to explore its direct effect on vascular cellular components. Here, we reported in vivo and in vitro that GLP-1R agonist liraglutide could protect against oxidized LDL-induced endothelial dysfunction, including enhancing vasodilatory response to acetylcholine, increasing NO bioavailability, and reducing oxidative stress, inflammation, and apoptosis, and the salutary effects of liraglutide were partly associated with GLP-1R dependent downregulation of the LOX-1/NOX4/NF-κB pathway.

In our in vivo experiments, we selected LDLR-KO mice fed a high-cholesterol diet as animal models to evaluate the damage of ox-LDL to vascular endothelium. First, we examined the endothelium-dependent vascular reactivity by organ chamber experiments and found that the acetylcholine-induced vasodilation of aortas from LDLR-KO mice was impaired compared with those from wild-type mice, which were significantly restored by liraglutide treatment to levels comparable to those of wild-type mice. However, the enhanced endothelium-dependent vasodilation by liraglutide was significantly blunted when GLP-1R antagonist exendin-9 was added. Then, we compared the circulatory levels of ox-LDL, oxidative stress and inflammatory markers, and NO between different groups, and showed that the plasma levels of ox-LDL in LDLR-KO mice were significantly higher than those in wild-type mice. Liraglutide treatment failed to reduce plasma ox-LDL levels in LDLR-KO mice, which is slightly different from the generally accepted moderate lipid-lowering effect of GLP-1R agonists [28] and may partly be ascribed to inadequate treatment duration of liraglutide. Although there was no significant change in ox-LDL, liraglutide reduced the elevation of oxidative stress and inflammatory markers and increased plasma NO levels in LDLR-KO mice, and these effects were attenuated with the addition of exendin-9. The expression of LOX-1 in the thoracic aorta by immunoblotting consistently showed that the inhibition of LOX-1 expression by liraglutide was eliminated by exendin-9. These results suggested that the protection of liraglutide against ox-LDL-associated endothelial dysfunction was partially dependent on GLP-1R activation and LOX-1 downregulation. Previous studies by other authors have also indicated the protective effect of liraglutide against endothelial dysfunction through other mechanisms. Gaspari et al. documented a GLP-1R-dependent inhibition of endothelial dysfunction by liraglutide in the ApoE^-/-^ mouse model through increasing eNOS expression and suppressing ICAM-1 expression in the aortic endothelium [13]. Wu et al. showed that liraglutide increased endothelial NO synthesis by the mTOR/Akt signaling pathway to alleviate endothelial dysfunction and induce an antiapoptotic effect [21]. Yue et al. reported that liraglutide alleviated the inflammatory response and reduced ox-LDL-induced endothelial dysfunction by upregulating the expression of Kruppel-like factor 2 [19]. However, none of these studies have elucidated the potential role of GLP-1R and LOX-1 in the protection of liraglutide against endothelial dysfunction.

To further elucidate the molecular mechanism by which liraglutide protects endothelial cells, we treated HUVECs with ox-LDL alone or in combination with liraglutide, in the presence or absence of LOX-1 overexpression or GLP-1R downregulation, and determined the protein expression of signaling modulators NOX4 and NF-κB. Consistent with the in vivo findings, our in vitro experiments showed that liraglutide significantly reduced the ox-LDL-induced apoptosis, ROS generation, and adhesion molecular expression, as well as the upregulated expression of LOX-1, NOX4, and NF-κB p65. When cells were pre-transfected with pcDNA3.1-LOX-1, the protective effect of liraglutide on ox-LDL-cultured endothelial cells and its inhibition on NOX4 and NF-κB p65 protein expression was partially weakened. These findings highlight the role of LOX-1 in ox-LDL-induced oxidative stress, inflammation, and endothelial apoptosis and the potential for liraglutide to protect endothelial function by targeting inhibition of the LOX-1/NOX4/NF-κB signaling pathway. In accordance with these results, previous studies have demonstrated that LOX-1 inhibition preserved endothelium-dependent vasodilation in two murine models of atherosclerosis [29,30]. However, there are only a few studies investigating the relationship between liraglutide and LOX-1 expression and the results are inconsistent. Dai et al. showed that liraglutide reduced the upregulated LOX-1 expression in ox-LDL-treated vascular smooth muscle cells [22], but did not affect LOX-1 expression in macrophages [31]. To further identify the relationship between GLP-1R activation and LOX-1 expression in the endothelium, we pre-transfected cells with siRNA against GLP-1R and found that the inhibitory effect of liraglutide on ox-LDL-induced LOX-1 protein upregulation was dependent on GLP-1R activation. Collectively, these in vitro findings suggest that liraglutide alleviates ox-LDL-induced endothelial dysfunction through GLP-1R-dependent down-regulation of the LOX-1/NOX4/NF-κB signaling pathway.

LOX-1 is the most important scavenger receptor in endothelial cells and macrophages for ox-LDL endocytosis. In addition, upon binding to ox-LDL, the activation of LOX-1-mediated oxidative stress and inflammation also contributes to endothelial dysfunction and atherosclerosis [32]. NOX4 is expressed in the endothelium at a higher level than other NADPH isoforms and is the main source of intracellular ROS generation [27,33]. Several studies have demonstrated an increased expression of NOX4 after LOX-1 activation [34–36]. Our in vitro experiments showed that both ox-LDL treatment and LOX-1 overexpression dramatically upregulated the expression of NOX4 in the endothelium, which was associated with increased ROS generation and NF-κB p65 protein expression. Liraglutide treatment reduced the ox-LDL-induced LOX-mediated protein expression of NOX4 and NF-κB p65. Previous studies have demonstrated that GLP-1R agonist inhibits NOX4-mediated ROS production in high-glucose cultured endothelial cells or angiotensin II-induced cardiac hypertrophy [37,38]. Our results further extend the possibility that GLP-1R agonist also alleviates non-diabetes-related oxidative stress and endothelial dysfunction, and elucidate the role of LOX-1 in mediating this process, suggesting that GLP-1R agonist may be used to reduce non-diabetes-related atherosclerotic events in the future.

There also exist some limitations in our study. First, we have not detected metabolic indices such as blood sugar levels and body weight except ox-LDL in our in vivo experiments, which are also considered as main pathways for liraglutide to confer cardiovascular benefits. Therefore, our study was unable to address whether the endothelial protective properties of liraglutide are independent of its hypoglycemic or weight reducing effects. Secondly, in addition to LOX-1, there are other scavenger receptors targeting ox-LDL on endothelial cells, such as SR-A and CD36, which were not detected in this study. Therefore, whether liraglutide may exert endothelial protection by acting on other scavenger receptors remains to be further explored in future studies. Thirdly, only one isoform of NADPH oxidase NOX4 was detected in this study. Although the latter is most expressed in endothelial cells, its effect on atherosclerosis is still controversial [39,40]. Therefore, future studies are warranted to investigate whether liraglutide can exert an antioxidant effect by inhibiting the expression of other NADPH oxidases.

**Figure 8.**
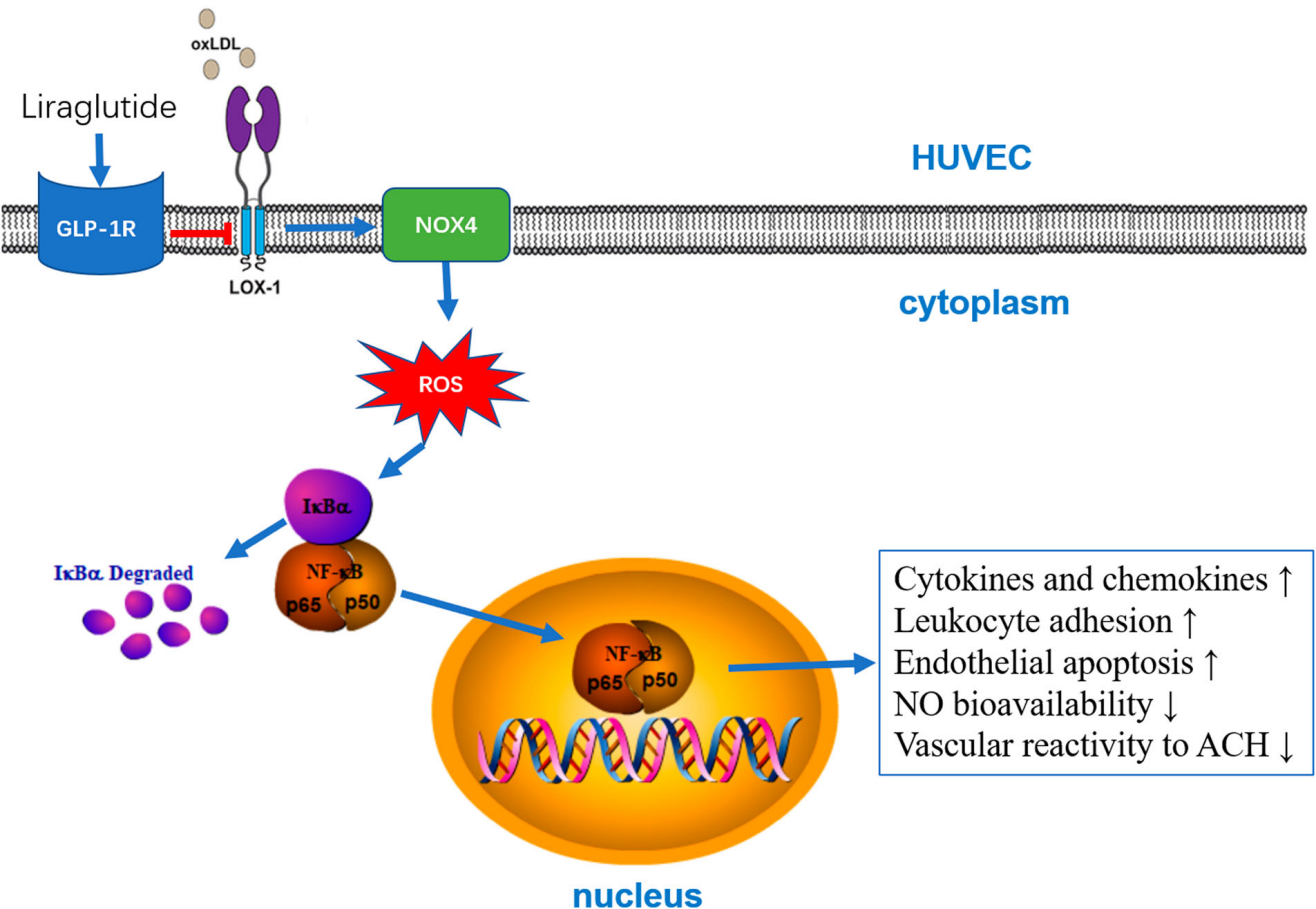
Schematic diagram illustrating the proposed signaling pathway involved in the protective effect of liraglutide against ox-LDL-associated endothelial dysfunction.

In summary, as depicted in Figure 8, our study showed that liraglutide effectively prevents oxidized LDL-induced oxidative stress, inflammation and endothelial apoptosis, increases NO bioavailability, and improves endothelium-dependent vasodilation, which is associated with GLP-1R-dependent inhibition of LOX-1-mediated NOX4/ NF-κB signaling.

## Data Availability

The data that support the findings of this study are available on request from the corresponding author.
